# Inefficient Toll-Like Receptor-4 Stimulation Enables *Bordetella parapertussis* to Avoid Host Immunity

**DOI:** 10.1371/journal.pone.0004280

**Published:** 2009-01-26

**Authors:** Daniel N. Wolfe, Anne M. Buboltz, Eric T. Harvill

**Affiliations:** 1 Department of Veterinary and Biomedical Sciences, The Pennsylvania State University, University Park, Pennsylvania, United States of America; 2 Graduate Program in Biochemistry, Microbiology, and Molecular Biology, The Pennsylvania State University, University Park, Pennsylvania, United States of America; Columbia University, United States of America

## Abstract

The recognition of bacterial lipopolysaccharide (LPS) by host Toll-like receptor (TLR)4 is a crucial step in developing protective immunity against several gram negative bacterial pathogens. *Bordetella bronchiseptica* and *B. pertussis* stimulate robust TLR4 responses that are required to control the infection, but a close relative, *B. parapertussis*, poorly stimulates this receptor, and TLR4 deficiency does not affect its course of infection. This led us to hypothesize that inefficient TLR4 stimulation enables *B. parapertussis* to evade host immunity. In a mouse model of infection, *B. parapertussis* grew rapidly in the lungs, but no measurable increase in TLR4-mediated cytokine, chemokine, or leukocyte responses were observed over the first few days of infection. Delivery of a TLR4 stimulant in the inoculum resulted in a robust inflammatory response and a 10- to 100-fold reduction of *B. parapertussis* numbers. As we have previously shown, *B. parapertussis* grows efficiently during the first week of infection even in animals passively immunized with antibodies. We show that this evasion of antibody-mediated clearance is dependent on the lack of TLR4 stimulation by *B. parapertussis* as co-inoculation with a TLR4 agonist resulted in 10,000-fold lower *B. parapertussis* numbers on day 3 in antibody-treated wild type, but not TLR4-deficient, mice. Together, these results indicate that inefficient TLR4 stimulation by *B. parapertussis* enables it to avoid host immunity and grow to high numbers in the respiratory tract of naïve and immunized hosts.

## Introduction

The ability of a pathogen to persist in its host for an extended period of time requires that it first evades rapid control and clearance by the innate immune response. Lipopolysaccharide (LPS), a major component of the outer membrane of gram negative bacteria, stimulates host Toll-like receptor (TLR)4 to initiate the production of pro-inflammatory cytokines and chemokines that recruit and activate leukocytes [1], [2], which is important to protection against many bacterial pathogens [3], [4], [5], [6], [7], [8]. Interestingly, LPS is not an invariant structure among gram negative bacteria. For example, *Salmonella* deacylates and palmitoylates lipid A in response to the host environment, allowing this bacterium to evade TLR4 responses [9], [10]. *Yersinia* and *Pseudomonas* species also modulate their LPS structures, resulting in diminished TLR4 responses to infection [11], [12], [13], [14]. These findings have led to the recent realization that bacteria can modulate pathogen associated molecular patterns, such as LPS, to optimize interactions with the host.

*Bordetella bronchiseptica*, *B. pertussis* and *B. parapertussis* are three very closely related species that make up the classical bordetellae. *Bordetella bronchiseptica*, infects a wide-range of mammals where it chronically colonizes the nasal cavity [15] and is often observed as a commensal [16], [17]. Both *B. pertussis* and *B. parapertussis* are highly infectious pathogens that cause the acute disease whooping cough in humans [16]. Each of these human-adapted species has independently evolved from a *B. bronchiseptica*-like progenitor [18], [19]. The comparative immunobiology of the bordetellae has shed light on some key differences among these bacteria. For example, the LPS structures of each of these bordetellae differs [20], [21], [22] which results in a wide-range of TLR4 responses and requirements [3]. The LPS of *B. pertussis* and *B. bronchiseptica* are very stimulatory of TLR4 and TLR4 is required for their clearance [3]. In contrast, the LPS of *B. parapertussis* LPS is much less stimulatory of TLR4 and TLR4-deficiency does not render mice more susceptible to *B. parapertussis* [3].

The clearance of these *Bordetella* species by antibodies also differs and appears to relate to their epidemiology [15]. *B. bronchiseptica* is rapidly cleared, three days post-inoculation, by adoptively transferred antibodies [15]. Previous studies have shown that this rapid antibody-mediated clearance is due to TLR4-dependent leukocyte recruitment [23]. *B. bronchiseptica* can persist for years within the nasal cavity of its host, where serum antibodies have no effect, and therefore a strong selection to avoid antibody-mediated clearance does not exist [15]. In contrast to *B. bronchiseptica*, *B. pertussis* and *B. parapertussis* avoid rapid antibody-mediated clearance for the first week of infection until a sufficient T-cell response is generated [15], [24]. Therefore, while both *B. pertussis* and *B. parapertussis* are more closely related to *B. bronchiseptica* than they are to each other [18], they share the ability to resist rapid antibody-mediated clearance from the lower respiratory tract. The high prevalence of detectable antibodies to *B. pertussis* and *B. parapertussis* in human populations, either due to vaccination or previous infection, presents a strong selection for the ability to avoid antibody-mediated clearance, allowing for repeated infection of individuals [15].

While both of these human-adapted species avoid rapid antibody-mediated clearance, they do so by distinct mechanisms. *B. pertussis* avoids rapid antibody-mediated clearance by inhibiting the TLR4-dependent recruitment of leukocytes to the lungs via pertussis toxin (Ptx) [25], [26]. A strain lacking Ptx (*B. pertussis*ΔPTx) is rapidly cleared from the lungs upon adoptive transfer of antibodies [25]. Since *B. parapertussis* lacks Ptx [27], this bacterium must avoid antibody-mediated clearance in a Ptx-independent manner.

Since the rapid antibody-mediated clearance of *B. bronchiseptica* is dependent on TLR4 [23] and *B. parapertussis* is a weak stimulator of TLR4 [3], we hypothesized that the inefficient TLR4 stimulation by *B. parapertussis* allows it to avoid the robust inflammatory response required for rapid antibody-mediated clearance. Using a mouse model of infection, we determined that co-inoculation of *B. parapertussis* with a TLR4 stimulant led to enhanced pro-inflammatory cytokine production and leukocyte accumulation as well as more efficient control and rapid antibody-mediated clearance of the bacteria. These results, observed in wild type but not TLR4-deficient animals, explain several characteristics of this important human pathogen and suggest interventions in the disease process. They also demonstrate how very closely related organisms can change complex structural components such as LPS to modulate stimulation of innate immune receptors to optimize their interactions with the host.

## Materials and Methods

### Bacterial strains and growth

*B. parapertussis* strain 12822 was isolated from German clinical trials [28] and 12822G is a gentamicin-resistant derivative of 12822 [24]. *B. bronchiseptica* strain RB50 was originally isolated from a rabbit [29]. Bacteria were maintained on Bordet-Gengou agar (Difco) containing 10% defibrinated sheep blood (Hema Resources) and appropriate antibiotics. Liquid culture bacteria were grown at 37°C overnight on a roller drum to mid-log phase in Stainer-Scholte broth.

### Inoculation of mice

C57BL/6, C3H/HEOuJ (wild type), and C3H/HEJ (TLR4-deficient) mice were obtained from Jackson Laboratories and bred in our *Bordetella*-free, specific pathogen-free facilities at The Pennsylvania State University. Bacteria grown overnight (to an optical density at 600 nm of approximately 0.3) in liquid culture were diluted in PBS to approximately 10^7^ CFU/ml. 50 µl of the inoculum (5×10^5^ CFU) was pipetted on to the external nares of 4–6 week old mice that had been lightly sedated with 5% isoflurane in oxygen. For co-inoculations with *B. parapertussis* and *B. bronchiseptica*, both species were present at 10^7^ CFU/ml in the inoculum and mice were inoculated as above (5×10^5^ CFU of each species in 50 µl). For co-inoculation with heat-killed *B. bronchiseptica*, bacteria were grown overnight to an optical density of 0.3 and heat-inactivated by incubating in a water bath at 75°C for 30 minutes. Bacteria in the inoculum were plated before and after heat-inactivation to quantify the number of bacteria present and ensure that the incubation killed *B. bronchiseptica*. Inocula were prepared so that they contained 10^7^ CFU/ml of *B. parapertussis* and 10^9^ CFU/ml of heat-killed *B. bronchiseptica* (5×10^5^ CFU *B. parapertussis* and 5×10^7^ CFU of heat-killed *B. bronchiseptica* in 50 µl per mouse). For co-inoculations with LPS, inocula were prepared containing 10^7^ CFU/ml of *B. parapertussis* and 10 µg/ml of purified LPS from *B. bronchiseptica*, *B. parapertussis*, or *E. coli* (5×10^5^ CFU *B. parapertussis* and 500 ng LPS in 50 µl per mouse). All protocols were reviewed by the university's Institutional Animal Care and Use Committee and all animals were handled in accordance with institutional guidelines.

### Adoptive transfer of serum antibodies

To generate convalescent phase (immune) serum, C57BL/6 mice were inoculated with 5×10^5^ CFU of *B. parapertussis* and allowed to convalesce for 28 days. By this time, these mice have generated high titers of *B. parapertussis*-specific antibodies [24]. Blood was then collected from these mice and the serum portion was isolated and stored at −80°C until use. 200 µl of immune serum was delivered by I.P. injection into mice immediately before inoculation. Serum from uninfected mice (naïve serum) was used as a control.

### Quantification of bacteria, leukocytes, and cytokines in the lungs

To quantify bacterial numbers, the lungs were excised on day 0, 0.5, 1, 2, 3, 7, or 14 post-inoculation. Lungs were homogenized in 1 ml of PBS. The lung homogenate was then plated onto Bordet-Gengou agar plates at the appropriate dilutions and CFU were counted 4 days later for *B. parapertussis* and 2 days later for *B. bronchiseptica*. To quantify leukocytes, mice were infected for 0, 0.5, 1, 2, 3, 7, or 14 days, sacrificed, and bronchoalveolar lavage (BAL) fluid was collected. Red blood cells were lysed by treatment with ammonium chloride as previously described [30]. Leukocytes were counted on a hemocytometer to quantify total numbers of leukocytes in the BAL fluid. Aliquots of cells were stained with FITC-labeled anti-Ly-6G (BD Biosciences Phramingen), and the percentage of Ly-6G positive cells was multiplied by the total number of leukocytes to calculate the number of neutrophils. For the quantification of cytokines and chemokines in the lungs, wild type or TLR4-deficient mice were inoculated with *B. parapertussis*, *B. bronchiseptica*, or both species and sacrificed 2 hours or 1 day later. Lungs were homogenized in 1 mL of PBS and samples were run on ELISAs specific for TNFα, KC, MIP-1α, and/or IL-1β according to the manufacturer's protocols (R&D Systems, Minneapolis, Minnesota, USA).

### *In vitro* growth curves of *B. parapertussis* and *B. bronchiseptica* and enumeration of co-inoculated samples

Both bacteria (RB50 and 12822G) were grown overnight to an optical density of 0.3. They were then diluted in fresh Stainer-Scholte broth to 10^7^ CFU/ml. The liquid cultures were then grown on a roller drum at 37°C and aliquots were plated at the indicated times on Bordet-Gengou agar plates with 20 µg/ml of streptomycin or gentamicin. The streptomycin plates, on which both species could have grown, were counted 2 days later, before *B. parapertussis* colonies became visible. The gentamicin plates, on which only *B. parapertussis* could grow, were counted 4 days later.

### Statistical Analysis

The mean+/−SD (error bars) was determined for CFU, leukocytes, and cytokines. For experiments quantifying bacterial numbers, either three or four mice were used per group. For all other experiments, four mice were used per group. Two-tailed, unpaired Student's T-tests were used to determine statistical significance between groups. All experiments were performed at least twice with similar results and *P*-values<0.05 were taken to be statistically significant.

## Results

### Reduction of *B. parapertussis* numbers correlates with an accumulation of leukocytes in the lungs

**Figure 1:**
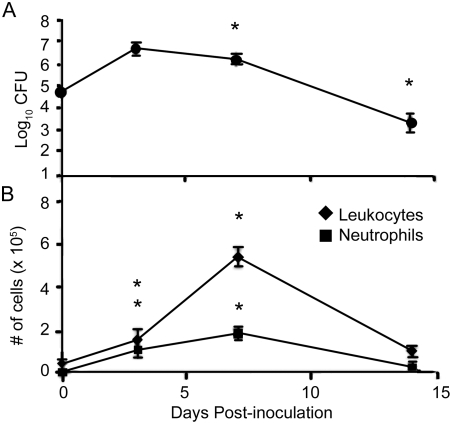
Numbers of *B. parapertussis* and leukocytes in the lungs over time. Groups of C57BL/6 mice were inoculated with *B. parapertussis* and sacrificed on day 0, 3, 7, or 14 post-inoculation. (A) Bacterial numbers in the lungs are represented as the Log_10_ mean+/−S.D. The dashed line represents the lower limit of detection. (B) Leukocyte and neutrophil numbers in the BAL fluid are represented as the mean+/−SD and asterisks denote P-values<0.05 when compared to numbers at day 3 for CFU (the highest observed numbers) or day 0 (naïve mice) for leukocytes.

*B. parapertussis* grows rapidly over the first few days post-inoculation but does not induce an early recruitment of neutrophils, which are known to be essential to eliminating this pathogen [3], [24]. Therefore, we sought to determine if the eventual reduction of *B. parapertussis* numbers in the lungs correlates with a delayed accumulation of neutrophils. C57BL/6 mice were inoculated with *B. parapertussis* and sacrificed on days 0, 3, 7, or 14 post-inoculation to quantify the numbers of bacteria in the lungs. *B. parapertussis* numbers peaked at approximately 4×10^6^ CFU on day 3, but began to decline by day 7 and were reduced to 6×10^3^ CFU by day 14 post-inoculation (Fig. 1A). Groups of C57BL/6 mice were also sacrificed on days 0, 3, 7, or 14 post-inoculation to quantify the numbers of leukocytes in the BAL fluid. Approximately 6×10^4^ leukocytes and less than 1×10^4^ neutrophils were recovered from the BAL fluid of uninfected mice. Leukocyte numbers had slightly, but significantly, increased by day 3 post-inoculation (1.6×10^5^ leukocytes, 1×10^5^ neutrophils), and peaked on day 7 post-inoculation (6×10^5^ leukocytes, 2×10^5^ neutrophils), declining thereafter (Fig. 1B). Together, these data show that the time when *B. parapertussis* numbers began to decline in murine lungs correlated with peak numbers of neutrophils in the lungs.

### *B. parapertussis* does not induce an early, TLR4-mediated leukocyte response

**Figure 2:**
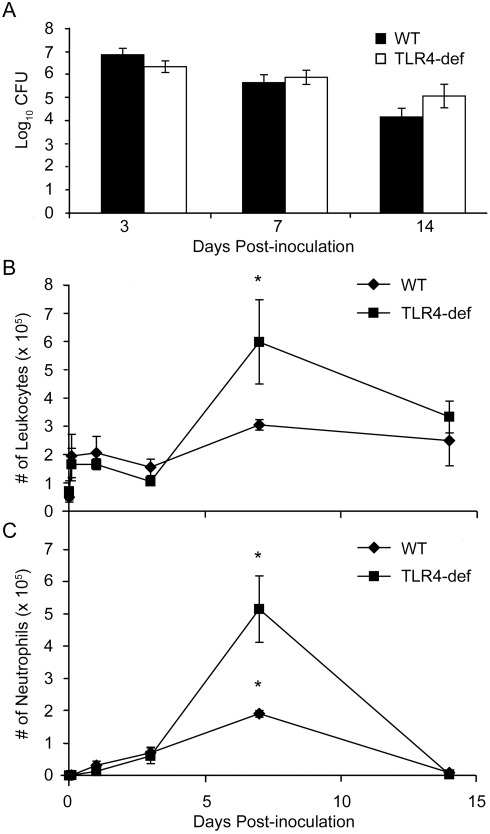
Numbers of leukocytes in the lungs of wild type and TLR4-deficient mice upon *B. parapertussis* infection. Groups of C3H/HEOuJ (WT) and C3H/HEJ (TLR4-def) mice were inoculated with *B. parapertussis* and sacrificed 2 hours later or on day 1, 3, 7, or 14 post-inoculation. (A) Bacterial numbers in the lungs were quantified on days 3, 7, and 14 post-inoculation and are expressed as the Log_10_ mean+/−SD. (B) Total leukocytes and (C) neutrophils in the BAL fluid were quantified at all time points and are represented as the mean+/−S.D. Asterisks denote P-values<0.05 when compared to naïve mice.

Although the induction of TLR4 responses is crucial to protection against other *Bordetella* species, *B. parapertussis* LPS does not efficiently stimulate these responses [3]. We addressed whether or not this pathogen induces any TLR4-dependent recruitment of leukocytes to the lungs over the course of infection. Wild type (C3H/HEOuJ) and TLR4-deficient (C3H/HEJ) mice were inoculated with *B. parapertussis* and sacrificed 0, 2 hours, 1, 3, 7, or 14 days later to quantify the numbers of bacteria in the lungs and leukocytes in the BAL fluid. As previously shown, similar bacterial numbers were observed in wild type and TLR4-deficient mice [3] (Fig. 2A). In the lungs of wild type mice, leukocyte numbers were highest on day 7 post-inoculation (∼3×10^5^ cells), and the same was true for TLR4-deficient mice (∼6×10^5^ cells) (Fig. 2B). Fewer than 10^5^ neutrophils were found in the lungs of both wild type and TLR4-deficient mice over the first 3 days post-inoculation but peaked on day 7 in both wild type (∼2×10^5^ cells) and TLR4-deficient (∼5×10^5^ cells) mice (Fig. 2C). Interestingly, more leukocytes accumulated in the lungs of TLR4-deficient mice compared to wild type mice. Therefore, TLR4 signaling does not measurably enhance the recruitment of leukocytes or the control of *B. parapertussis* infection, but may affect anti-inflammatory signals in response to this bacterium.

### TLR4-mediated cytokine and chemokine responses are not inhibited by *B. parapertussis* during infection of mice

**Figure 3:**
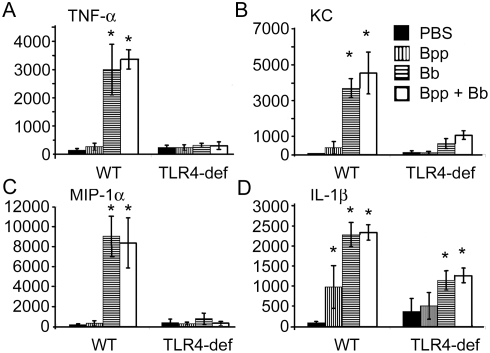
Effect of a co-inoculation with *B. parapertussis* on the TLR4-mediated cytokine response to *B. bronchiseptica*. Groups of wild type C3H/HEOuJ and TLR4-deficient C3H/HEJ mice were inoculated with *B. parapertussis* (Bpp), *B. bronchiseptica* (Bb), or both bacteria (Bpp+Bb) and sacrificed 2 hours later for the quantification of (A) TNF-α, (B) KC, (C) MIP-1α, or (D) IL-1β in lungs homogenized in 1 mL of PBS. Cytokine and chemokine numbers are expressed as the mean+/−SD. Asterisks represent P-values<0.05 when compared to mock-infected mice.

The lack of a measurable TLR4-mediated accumulation of leukocytes in response to *B. parapertussis* (Fig. 2) led us to assess whether or not *B. parapertussis* actively inhibits TLR4-mediated cytokine production. For these experiments, the effects of *B. parapertussis* on TLR4-mediated responses to a respiratory pathogen that is closely related and a potent stimulator of TLR4, *B. bronchiseptica*, were examined. Wild type and TLR4-deficient mice were inoculated with *B. parapertussis*, *B. bronchiseptica*, or both bacteria and sacrificed 2 hours later. *B. parapertussis* did not induce significant levels of TNF-α, KC, or MIP-1α in wild type or TLR4-deficient mice relative to mock-infected controls (Fig. 3A–C). Approximately 1000 pg of IL-1β was produced in the lungs of wild type mice in response to *B. parapertussis*, but this was not significantly different from the amount produced by TLR4-deficient mice (Fig. 3D). *B. bronchiseptica* induced the production of approximately 3000 pg of TNF-α, 3500 pg of KC, 9000 pg of MIP-1α, and 2300 pg of IL-1β in the lungs of wild type mice, but much lower levels in TLR4-deficient mice (250, 125, 200, and 1200 pg respectively) (Fig. 3A–D). Similar to *B. bronchiseptica*, the lungs of wild type mice that were inoculated with both species contained approximately 3500 pg of TNF-α,4500 pg of KC, 8500 pg of MIP-1α, and 2400 pg of IL-1β, and this production was also dependent on TLR4 (Fig. 3A–D). Together, these data indicate that *B. parapertussis* does not stimulate TLR4 or inhibit the TLR4-mediated cytokine and chemokine responses to *B. bronchiseptica* infection.

### Co-inoculation with *B. bronchiseptica* allows for more efficient control of *B. parapertussis*

**Figure 4:**
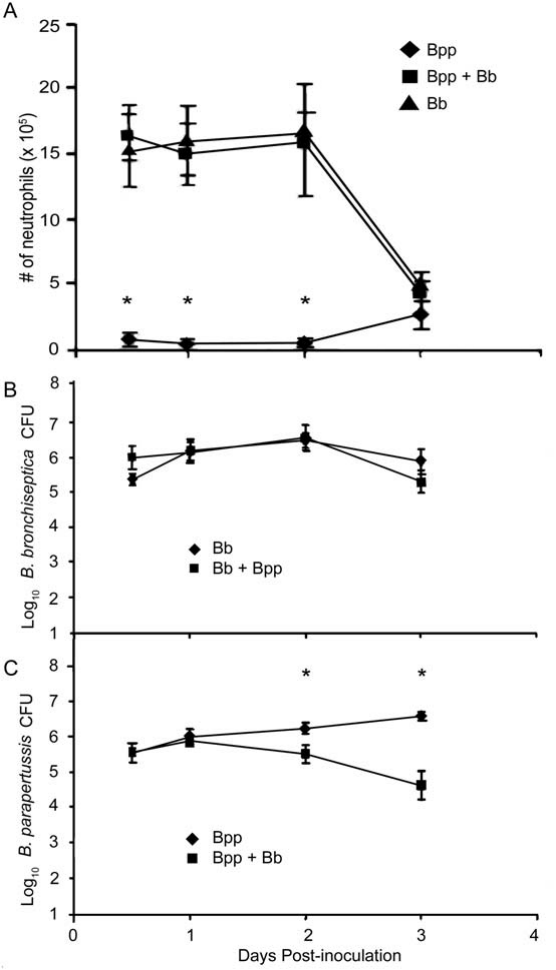
*In vivo* growth of *B. bronchiseptica* on *B. parapertussis* upon a co-inoculation. (A) Groups of C57BL/6 mice were inoculated with *B. parapertussis* (Bpp), *B. bronchiseptica* (Bb), or both bacteria (Bpp+Bb) and sacrificed 0.5, 1, 2, or 3 days later to quantify neutrophils in the BAL fluid. Groups of mice were also sacrificed at these times to quantify (B) Bb numbers and (C) Bpp numbers in the lungs. Neutrophil numbers are expressed as the mean+/−SD and bacterial numbers are expressed as the Log_10_ mean+/−SD. Asterisks represent P-values<0.05 when comparing *B. parapertussis*-infected mice to co-infected mice.

The robust, TLR4-mediated cytokine and chemokine responses to a co-inoculation with *B. parapertussis* and *B. bronchiseptica* led us to examine the effect of the co-inoculation on the accumulation of leukocytes and clearance of these bacteria. Wild type mice were inoculated with *B. parapertussis*, *B. bronchiseptica*, or both species and sacrificed 12 hours, 1, 2, or 3 days later to quantify neutrophils in the BAL fluid. Consistent with Figures 1 and 2, the BAL fluid of *B. parapertussis*-infected mice contained few neutrophils (<10^5^/ml of BAL fluid) over the first three days post-inoculation (Fig. 4A). In contrast, *B. bronchiseptica* induced the accumulation of approximately 1.5×10^6^ neutrophils/ml of BAL fluid over the first two days. This early recruitment of neutrophils to the lungs upon *B. bronchiseptica* infection is dependent on TLR4 [3]. The BAL fluid of co-inoculated mice also contained approximately 1.5×10^6^ neutrophils/ml for the first two days (Fig. 4A), indicating that *B. parapertussis* did not measurably inhibit the TLR4-mediated recruitment of neutrophils to the lungs in response to *B. bronchiseptica* infection.

Groups of C57BL/6 mice were then inoculated with *B. parapertussis*, *B. bronchiseptica*, or both bacteria and sacrificed 12 hours, 1, 2, or 3 days later to quantify bacterial numbers in the lungs. *B. bronchiseptica* numbers were not affected by a co-inoculation with *B. parapertussis* (Fig. 4B). When inoculated alone, *B. parapertussis* numbers rose over the first three days, peaking at approximately 3×10^6^ CFU on day 3 post-inoculation. When co-inoculated with *B. bronchiseptica*, however, *B. parapertussis* numbers began to decline after one day and were reduced to approximately 5×10^4^ CFU by day 3 post-inoculation, a 99% reduction from numbers of *B. parapertussis* alone (Fig. 4C). Together, these data indicate that a co-infection with *B. bronchiseptica* results in increased neutrophil recruitment and more efficient control of *B. parapertussis*.

To determine if *B. parapertussis* and *B. bronchiseptica* directly affected the growth of one another, they were grown together in liquid culture. *B. bronchiseptica* grew from approximately 10^7^ CFU/ml to 10^11^ CFU/ml in 24 hours and its growth was not affected by a co-inoculation with *B. parapertussis* (data not shown). *B. parapertussis*, which grows slower than *B. bronchiseptica*, grew from approximately 10^7^ CFU/ml to 10^10^ CFU/ml in 24 hours and its growth rate was not affected by a co-inoculation with *B. bronchiseptica* (data not shown). Thus, *B. parapertussis* and *B. bronchiseptica* do not directly affect each other's growth, even when grown to high density *in vitro*.

### Co-inoculation with *B. bronchiseptica* results in rapid antibody-mediated clearance of *B. parapertussis*

**Figure 5:**
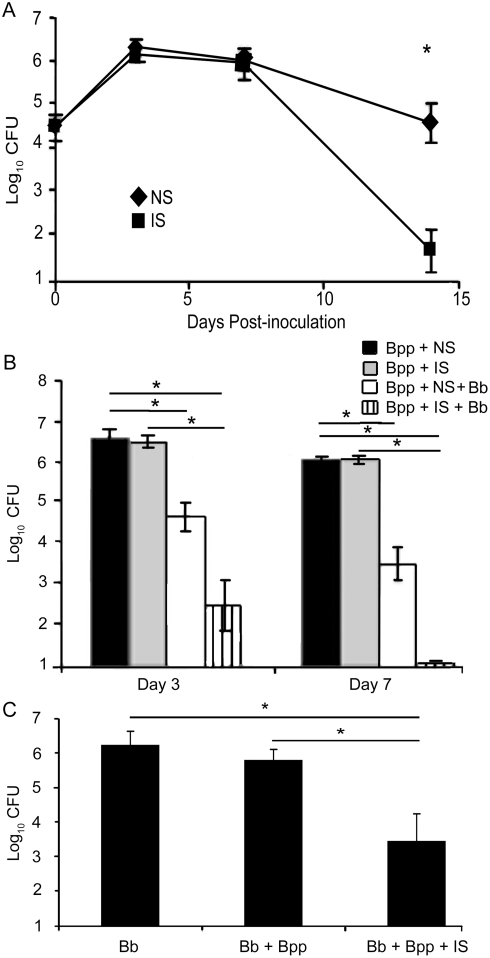
Antibody-mediated clearance of *B. parapertussis* upon a co-inoculation with *B. bronchiseptica*. (A) C57BL/6 mice were inoculated with *B. parapertussis*, given an adoptive transfer of naïve serum (NS) or immune serum (IS), and sacrificed 0, 3, 7, or 14 days later to quantify bacterial numbers in the lungs. (B–C) C57BL/6 mice were inoculated with *B. parapertussis* (Bpp), *B. bronchiseptica* (Bb), or both bacteria (Bpp+Bb), given an adoptive transfer of naïve serum (NS) or immune serum (IS), and sacrificed 3 or 7 days later. Numbers of (B) *B. parapertussis* and (C) *B. bronchiseptica* in the lungs were quantified. Bacterial numbers are expressed as the Log_10_ mean+/−SD. Asterisks represent P-values<0.05.

*B. bronchiseptica* is cleared by antibodies within about three days via a TLR4-dependent mechanism [23]. In contrast, antibodies have no effect on the course of *B. parapertussis* infection during the first week but eliminate the infection during the second week [15], [24] (Fig. 5A), after significant numbers of neutrophils have accumulated in the lungs. Thus, we hypothesized that the lack of an early TLR4-mediated neutrophil recruitment allows *B. parapertussis* to delay antibody-mediated clearance. To test this, we examined the effect of stimulating TLR4 responses on the rapid antibody-mediated clearance of *B. parapertussis* by inoculating mice with one species or both species and giving an I.P. injection of naïve serum or convalescent phase (immune) serum. Groups of mice were then sacrificed on day 3 or 7 post-inoculation for the enumeration of bacteria in the lungs. While immune serum alone had no measurable effect on the numbers of *B. parapertussis* [15] (Fig. 5B), immune serum with a co-inoculation of *B. bronchiseptica* rapidly reduced *B. parapertussis* numbers >99%, to approximately 100 CFU by day 3 and to undetectable levels by day 7 post-inoculation (Fig. 5B). These data indicate that a co-inoculation with *B. bronchiseptica* results in rapid antibody-mediated clearance of *B. parapertussis*. The co-inoculation did not affect the ability of *B. bronchiseptica* to colonize the lungs of mice treated with naïve serum, but *B. bronchiseptica* numbers were approximately 500-fold lower in the lungs of mice treated with immune serum (Fig. 5C). This was likely due to *B. parapertussis*-induced antibodies being cross reactive with *B. bronchiseptica* antigens.

### Rapid clearance of *B. parapertussis* upon co-inoculation with *B. bronchiseptica* is dependent on TLR4

**Figure 6:**
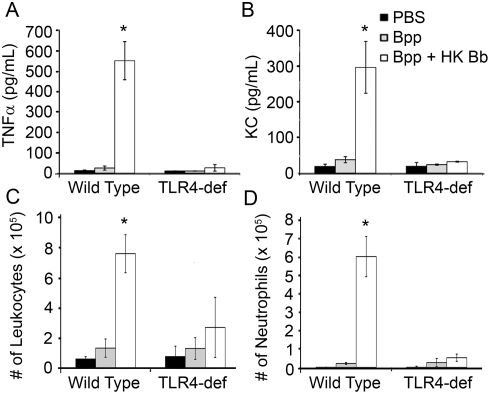
Cytokine and leukocyte levels in the lungs of mice infected with *B. parapertussis* and heat-killed *B. bronchiseptica*. Groups of wild type (C3H/HEOuJ) and TLR4-deficient (C3H/HEJ) mice were inoculated with PBS, *B. parapertussis* (Bpp) or Bpp and heat-killed *B. bronchiseptica* (HK Bb). (A) TNFα, (B) KC, (C) leukocyte and (D) neutrophil levels were quantified in the BAL fluid one day later. Cytokine levels and cell numbers are represented as the mean+/−SD. Asterisks represent P-values<0.05 when compared to mock-infected mice.

We hypothesized that the protective effects of adding *B. bronchiseptica* to the *B. parapertussis* inoculum were due to the robust TLR4-mediated inflammatory response to *B. bronchiseptica*. To test this, groups of wild type and TLR4-deficient mice were inoculated with *B. parapertussis* alone or *B. parapertussis* with heat-killed *B. bronchiseptica*. Heat-killed *B. bronchiseptica* was used because live *B. bronchiseptica* is lethal to TLR4-deficient mice within approximately 3 days [3]. This also allowed us to address whether or not the effect on *B. parapertussis* numbers required live *B. bronchiseptica*, or if stimulation of the immune response by heat-inactivated components was sufficient to reduce bacterial numbers. The cytokine and leukocyte responses were measured 1 day post-inoculation with *B. parapertussis* alone versus *B. parapertussis* with heat-killed *B. bronchiseptica*. Inoculation with *B. parapertussis* did not induce levels of TNFα and KC in the BAL fluid of wild type or TLR4-deficient mice that were measurably different from mock-infected lungs (Fig. 6A–B). In contrast, co-inoculation with *B. parapertussis* and heat-killed *B. bronchiseptica* resulted in high levels of TLR4-dependent TNFα and KC production (approximately 550 and 300 pg respectively). When leukocyte numbers were examined, *B. parapertussis* alone did not induce significant levels of leukocyte accumulation (∼1×10^5^ leukocytes, ∼2×10^4^ neutrophils) relative to mock-infected mice (Fig. 6C–D). Co-inoculation with heat-killed *B. bronchiseptica*, however, resulted in the accumulation of 8×10^5^ leukocytes and 6×10^5^ neutrophils in the lungs of wild type mice by this time, while the lungs of TLR4-deficient mice contained only 3×10^5^ leukocytes and 4×10^4^ neutrophils (Fig. 6C–D). Thus, heat-killed *B. bronchiseptica* induced robust, TLR4-mediated cytokine and leukocyte responses.

**Figure 7:**
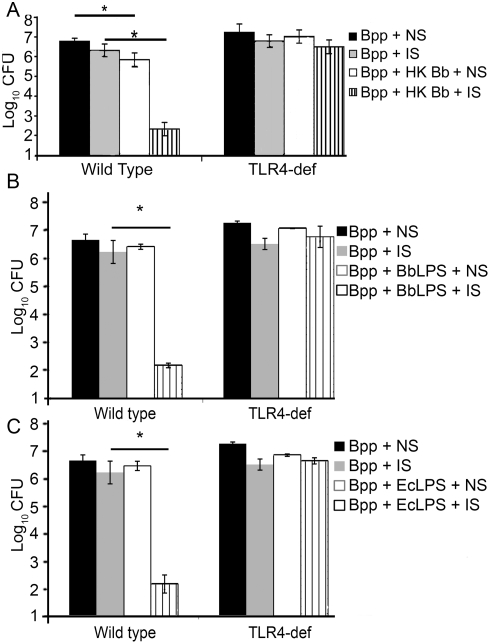
Effect of TLR4 stimulation on the rapid antibody-mediated clearance of *B. parapertussis*. (A) Groups of wild type (C3H/HEOuJ) and TLR4-deficient (C3H/HEJ) mice were inoculated with *B. parapertussis* (Bpp) or Bpp and heat-killed *B. bronchiseptica* (HK Bb) and given I.P. injections of naïve serum (NS) or immune serum (IS). Groups of mice were also inoculated with Bpp and (B) Bb LPS (BbLPS) or (C) *E. coli* LPS (EcLPS) and treated with naïve or immune serum. Bacterial numbers were quantified 3 days later and are expressed as the Log_10_ mean+/−SD. Asterisks denote P-values<0.05.

To address the effect on bacterial numbers, wild type and TLR4-deficient mice were then inoculated with *B. parapertussis* alone or *B. parapertussis* with heat-killed *B. bronchiseptica*. These mice were also given an I.P. injection of naïve serum or immune serum and sacrificed 3 days later. In wild type mice that were treated with naïve serum, co-inoculation with heat-killed *B. bronchiseptica* resulted in a 10-fold reduction of *B. parapertussis* numbers. In wild type mice that were treated with immune serum, the co-inoculation resulted in *B. parapertussis* numbers being reduced to nearly undetectable levels within 3 days (Fig. 7A). In TLR4-deficient mice, however, the co-inoculation had no effect on *B. parapertussis* numbers in either naïve serum treated or immune serum treated mice (Fig. 7A). Wild type and TLR4-deficient mice were then inoculated with *B. parapertussis* and purified LPS from *B. bronchiseptica*, *E. coli*, or *B. parapertussis* to determine if the addition of TLR4 stimulatory LPS was the key to rapid clearance of *B. parapertussis*. Co-inoculation with *B. bronchiseptica* LPS resulted in a 10,000-fold reduction in bacterial numbers in the lungs of wild type mice treated with immune serum, but did not affect bacterial numbers in TLR4-deficient mice (Fig. 7B). Similar results were observed when mice were co-inoculated with LPS from *E. coli* (Fig. 7C). In contrast, co-inoculation with purified LPS from *B. parapertussis*, a weak TLR4-stimulant [3], had no effect on *B. parapertussis* numbers in the lungs of wild type or TLR4-deficient mice (data not shown). Thus, TLR4 was required for the enhanced clearance of *B. parapertussis* upon co-inoculation with strong TLR4 stimulants (Fig. 7B–C). Combined, these data suggest that a lack of TLR4 stimulation enables *B. parapertussis* to avoid immune clearance and grow to higher numbers within the host.

## Discussion

*B. parapertussis* is able to delay clearance by avoiding the induction of a robust innate immune response. Here, we show that its slow clearance from murine lungs correlates with the accumulation of neutrophils in these lungs, which is delayed in comparison to the neutrophil responses to other closely related bacteria (Mann, Harvill, unpublished data). We predicted that inefficient TLR4 stimulation by *B. parapertussis* LPS may result in the low level of neutrophil accumulation in response to infection over the first few days and may allow this pathogen to grow rapidly during this time, even in animals given a passive transfer of immune serum. In support of this, co-inoculation with a potent stimulator of TLR4 resulted in enhanced control and rapid antibody-mediated clearance of *B. parapertussis* from wild type, but not TLR4-deficient mice. This study provides an example of how expressing an LPS that is a poor stimulator of TLR4 can facilitate persistence of a gram negative bacterium within its host.

LPS modulation is often utilized by gram negative bacterial pathogens to optimize interactions with host immunity. For example, *Yersinia pestis* produces a TLR4-stimulatory LPS at 26°C, but an unstimulatory LPS at 37°C, the body temperature of its typical mammalian host [11], [12], [13]. Montminy et.al. genetically modified *Y. pestis* so that it would produce the stimulatory 26°C LPS at all times [31]. While wild type *Y. pestis* causes sepsis and mortality in a mouse model of infection, the expression of TLR4-stimulatory LPS resulted in containment of the infection by the innate immune response and less efficient systemic spread of the infection [31]. Similarly, co-inoculation of *B. parapertussis* with a TLR4 agonist resulted in an attenuated course of infection (Fig. 4, Fig. 7). The expression of LPS molecules that poorly stimulate TLR4 appears to hinder the generation of effective immunity against *Y. pestis* [31] and *B. parapertussis*, and may be a stealth strategy shared by other gram negative bacteria as well [32], [33], [34], [35].

TLR4 stimulation by LPS results in a branched downstream signaling pathway consisting of a Mal/MyD88 branch and a TRIF/TRAM branch that leads to the production of several different pro-inflammatory cytokines [36]. However, each branch is crucial to the production of a different subset of cytokines. For example, TNF-α and CCL3 are MyD88-dependent cytokines while IFN-β is a TRIF-dependent cytokine [37], [38]. Although our *in vivo* data presented above suggested that *B. parapertussis* does not induce measurable amounts of TLR4-mediated cytokine production or leukocyte recruitment (Fig.3, Fig. 6), the higher numbers of leukocytes in TLR4-deficient mice suggests that leukocyte responses to *B. parapertussis* infection may be limited by a TLR4-dependent mechanism. *B. parapertussis* may upregulate the TRIF/TRAM branch of TLR4 signaling, as this branch appears to play a role in endotoxin tolerance [39]. We have also recently observed that IL-10 dampens the inflammatory response to *B. parapertussis in vivo* and induces the production of IL-10 *in vitro* (Wolfe and Hester, unpublished data). Since IL-10 production can be induced by TLR4 stimulation [4], it is reasonable to suggest that the anti-inflammatory effect of TLR4 in Figure 2 may be mediated by IL-10.

In contrast to *B. bronchiseptica*, passively transferred antibodies have no effect on colonization by the human pathogens *B. parapertussis* and *B. pertussis* over the first week of infection [15], [24], [25]. This is likely important to the success of these pathogens, as they are able to re-infect the same host multiple times despite a measurable antibody response [40]. Our lab previously showed that Ptx delays antibody-mediated clearance of *B. pertussis* by inhibiting the migration of neutrophils to the lungs [25]. Although *B. parapertussis* does not express Ptx, poor induction of TLR4 signaling appears to be an alternative method for limiting the neutrophil response and delaying antibody-mediated clearance. Limiting and/or inhibiting pro-inflammatory TLR4 stimulation may be crucial to the ability of *B. parapertussis* to remain endemic in human populations.

In addition to the inefficient stimulation of pro-inflammatory TLR4 responses [3], likely due to its lipid A structure, the O-antigen of *B. parapertussis* LPS also appears to allow it to avoid rapid clearance by antibodies induce by *B. pertussis* infection or vaccination [24]. O-antigen prevents the binding of *B. pertussis*-induced antibodies to the surface of *B. parapertussis*, allowing the latter to colonize hosts that had been previously immunized against the former. This provides an example of a single molecule, LPS, providing multiple, non-overlapping mechanisms to protect a bacterium against the effects of antibodies.

Despite excellent vaccine coverage, whooping cough has been re-emerging in vaccinated populations [41], [42], [43], [44], [45], but it is unclear what the relative roles of *B. pertussis* and *B. parapertussis* are in this resurgence [46]. Importantly, immunity induced by current vaccines protects against *B. pertussis* disease, but is largely ineffective against *B. parapertussis* disease [47], [48], [49], [50], [51]. The widespread use of vaccines appears to have resulted in a higher incidence of *B. parapertussis* as the causative agent of whooping cough in vaccinated individuals relative to unvaccinated individuals [49]. Current acellular vaccines induce a T cell response that is Th2-skewed [52]. Given that our data shows that the clearance of *B. parapertussis* by antibodies is enhanced by pro-inflammatory responses, a vaccine that generates a strong Th1-skewed response to *B. parapertussis*, as opposed to a partially cross-reactive Th2 type response, could potentially provide more efficient protection against this pathogen.

## References

[1] Raetz CR, Whitfield C (2002). Lipopolysaccharide endotoxins.. Annu Rev Biochem.

[2] Lien E, Means TK, Heine H, Yoshimura A, Kusumoto S (2000). Toll-like receptor 4 imparts ligand-specific recognition of bacterial lipopolysaccharide.. J Clin Invest.

[3] Mann PB, Wolfe D, Latz E, Golenbock D, Preston A (2005). Comparative toll-like receptor 4-mediated innate host defense to Bordetella infection.. Infect Immun.

[4] Higgins SC, Lavelle EC, McCann C, Keogh B, McNeela E (2003). Toll-like receptor 4-mediated innate IL-10 activates antigen-specific regulatory T cells and confers resistance to Bordetella pertussis by inhibiting inflammatory pathology.. J Immunol.

[5] Schurr JR, Young E, Byrne P, Steele C, Shellito JE (2005). Central role of toll-like receptor 4 signaling and host defense in experimental pneumonia caused by Gram-negative bacteria.. Infect Immun.

[6] Abel B, Thieblemont N, Quesniaux VJ, Brown N, Mpagi J (2002). Toll-like receptor 4 expression is required to control chronic Mycobacterium tuberculosis infection in mice.. J Immunol.

[7] Supajatura V, Ushio H, Nakao A, Okumura K, Ra C (2001). Protective roles of mast cells against enterobacterial infection are mediated by Toll-like receptor 4.. J Immunol.

[8] Bernheiden M, Heinrich JM, Minigo G, Schutt C, Stelter F (2001). LBP, CD14, TLR4 and the murine innate immune response to a peritoneal Salmonella infection.. J Endotoxin Res.

[9] Kawasaki K, Ernst RK, Miller SI (2004). 3-O-deacylation of lipid A by PagL, a PhoP/PhoQ-regulated deacylase of Salmonella typhimurium, modulates signaling through Toll-like receptor 4.. J Biol Chem.

[10] Kawasaki K, Ernst RK, Miller SI (2004). Deacylation and palmitoylation of lipid A by Salmonellae outer membrane enzymes modulate host signaling through Toll-like receptor 4.. J Endotoxin Res.

[11] Kawahara K, Tsukano H, Watanabe H, Lindner B, Matsuura M (2002). Modification of the structure and activity of lipid A in Yersinia pestis lipopolysaccharide by growth temperature.. Infect Immun.

[12] Knirel YA, Lindner B, Vinogradov EV, Kocharova NA, Senchenkova SN (2005). Temperature-dependent variations and intraspecies diversity of the structure of the lipopolysaccharide of Yersinia pestis.. Biochemistry.

[13] Rebeil R, Ernst RK, Gowen BB, Miller SI, Hinnebusch BJ (2004). Variation in lipid A structure in the pathogenic yersiniae.. Mol Microbiol.

[14] Ernst RK, Adams KN, Moskowitz SM, Kraig GM, Kawasaki K (2006). The Pseudomonas aeruginosa lipid A deacylase: selection for expression and loss within the cystic fibrosis airway.. J Bacteriol.

[15] Kirimanjeswara GS, Mann PB, Harvill ET (2003). Role of antibodies in immunity to Bordetella infections.. Infect Immun.

[16] Mattoo S, Cherry JD (2005). Molecular pathogenesis, epidemiology, and clinical manifestations of respiratory infections due to Bordetella pertussis and other Bordetella subspecies.. Clin Microbiol Rev.

[17] Goodnow RA (1980). Biology of Bordetella bronchiseptica.. Microbiol Rev.

[18] Parkhill J, Sebaihia M, Preston A, Murphy LD, Thomson N (2003). Comparative analysis of the genome sequences of Bordetella pertussis, Bordetella parapertussis and Bordetella bronchiseptica.. Nat Genet.

[19] van der Zee A, Mooi F, Van Embden J, Musser J (1997). Molecular evolution and host adaptation of Bordetella spp.: phylogenetic analysis using multilocus enzyme electrophoresis and typing with three insertion sequences.. J Bacteriol.

[20] Preston A, Petersen BO, Duus JO, Kubler-Kielb J, Ben-Menachem G (2006). Complete structures of Bordetella bronchiseptica and Bordetella parapertussis lipopolysaccharides.. J Biol Chem.

[21] van den Akker WM (1998). Lipopolysaccharide expression within the genus Bordetella: influence of temperature and phase variation.. Microbiology.

[22] Caroff M, Aussel L, Zarrouk H, Martin A, Richards JC (2001). Structural variability and originality of the Bordetella endotoxins.. J Endotoxin Res.

[23] Kirimanjeswara GS, Mann PB, Pilione M, Kennett MJ, Harvill ET (2005). The complex mechanism of antibody-mediated clearance of Bordetella from the lungs requires TLR4.. J Immunol.

[24] Wolfe DN, Kirimanjeswara GS, Harvill ET (2005). Clearance of Bordetella parapertussis from the lower respiratory tract requires humoral and cellular immunity.. Infect Immun.

[25] Kirimanjeswara GS, Agosto LM, Kennett MJ, Bjornstad ON, Harvill ET (2005). Pertussis toxin inhibits neutrophil recruitment to delay antibody-mediated clearance of Bordetella pertussis.. J Clin Invest.

[26] Carbonetti NH, Artamonova GV, Andreasen C, Bushar N (2005). Pertussis toxin and adenylate cyclase toxin provide a one-two punch for establishment of Bordetella pertussis infection of the respiratory tract.. Infect Immun.

[27] Arico B, Rappuoli R (1987). Bordetella parapertussis and Bordetella bronchiseptica contain transcriptionally silent pertussis toxin genes.. J Bacteriol.

[28] Heininger U, Cotter PA, Fescemyer HW, Martinez de Tejada G, Yuk MH (2002). Comparative phenotypic analysis of the Bordetella parapertussis isolate chosen for genomic sequencing.. Infect Immun.

[29] Cotter PA, Miller JF (1994). BvgAS-mediated signal transduction: analysis of phase-locked regulatory mutants of Bordetella bronchiseptica in a rabbit model.. Infect Immun.

[30] Pilione MR, Harvill ET (2006). The Bordetella bronchiseptica type III secretion system inhibits gamma interferon production that is required for efficient antibody-mediated bacterial clearance.. Infect Immun.

[31] Montminy SW, Khan N, McGrath S, Walkowicz MJ, Sharp F (2006). Virulence factors of Yersinia pestis are overcome by a strong lipopolysaccharide response.. Nat Immunol.

[32] Trent MS, Stead CM, Tran AX, Hankins JV (2006). Diversity of endotoxin and its impact on pathogenesis.. J Endotoxin Res.

[33] Hajjar AM, Harvey MD, Shaffer SA, Goodlett DR, Sjostedt A (2006). Lack of in vitro and in vivo recognition of Francisella tularensis subspecies lipopolysaccharide by Toll-like receptors.. Infect Immun.

[34] Dixon DR, Darveau RP (2005). Lipopolysaccharide heterogeneity: innate host responses to bacterial modification of lipid a structure.. J Dent Res.

[35] Barquero-Calvo E, Chaves-Olarte E, Weiss DS, Guzman-Verri C, Chacon-Diaz C (2007). Brucella abortus uses a stealthy strategy to avoid activation of the innate immune system during the onset of infection.. PLoS ONE.

[36] Palsson-McDermott EM, O'Neill LA (2004). Signal transduction by the lipopolysaccharide receptor, Toll-like receptor-4.. Immunology.

[37] Kielian T, Phulwani NK, Esen N, Syed MM, Haney AC (2007). MyD88-dependent signals are essential for the host immune response in experimental brain abscess.. J Immunol.

[38] Lee JY, Lowell CA, Lemay DG, Youn HS, Rhee SH (2005). The regulation of the expression of inducible nitric oxide synthase by Src-family tyrosine kinases mediated through MyD88-independent signaling pathways of Toll-like receptor 4.. Biochem Pharmacol.

[39] Biswas SK, Bist P, Dhillon MK, Kajiji T, Del Fresno C (2007). Role for MyD88-independent, TRIF pathway in lipid A/TLR4-induced endotoxin tolerance.. J Immunol.

[40] Cherry JD, Heininger U, Feigin JDCRD, Demmler GJ, Kaplan S (2004). Pertussis and other *Bordetella* infections.. Textbook of pediatric infectious diseases, 5th ed..

[41] Celentano LP, Massari M, Paramatti D, Salmaso S, Tozzi AE (2005). Resurgence of pertussis in Europe.. Pediatr Infect Dis J.

[42] Center for Disease Control and Prevention (2002). Pertussis–United States, 1997–2000.. JAMA.

[43] von Konig CH, Halperin S, Riffelmann M, Guiso N (2002). Pertussis of adults and infants.. Lancet Infect Dis.

[44] Skowronski DM, De Serres G, MacDonald D, Wu W, Shaw C (2002). The changing age and seasonal profile of pertussis in Canada.. J Infect Dis.

[45] de Melker HE, Schellekens JF, Neppelenbroek SE, Mooi FR, Rumke HC (2000). Reemergence of pertussis in the highly vaccinated population of the Netherlands: observations on surveillance data.. Emerg Infect Dis.

[46] Watanabe M, Nagai M (2004). Whooping cough due to Bordetella parapertussis: an unresolved problem.. Expert Rev Anti Infect Ther.

[47] Heininger U, Stehr K, Cherry JD (1998). The efficacy of a whole cell pertussis vaccine and fimbriae against Bordetella pertussis and Bordetella parapertussis infections in respiratory mouse model.. Vaccine.

[48] Willems RJ, Kamerbeek J, Geuijen CA, Top J, Gielen H (1998). The efficacy of a whole cell pertussis vaccine and fimbriae against Bordetella pertussis and Bordetella parapertussis infections in a respiratory mouse model.. Vaccine.

[49] Liese JG, Renner C, Stojanov S, Belohradsky BH (2003). Clinical and epidemiological picture of B pertussis and B parapertussis infections after introduction of acellular pertussis vaccines.. Arch Dis Child.

[50] Stehr K, Cherry JD, Heininger U, Schmitt-Grohe S, uberall M (1998). A comparative efficacy trial in Germany in infants who received either the Lederle/Takeda acellular pertussis component DTP (DTaP) vaccine, the Lederle whole-cell component DTP vaccine, or DT vaccine.. Pediatrics.

[51] David S, van Furth R, Mooi FR (2004). Efficacies of whole cell and acellular pertussis vaccines against Bordetella parapertussis in a mouse model.. Vaccine.

[52] Barnard A, Mahon BP, Watkins J, Redhead K, Mills KH (1996). Th1/Th2 cell dichotomy in acquired immunity to Bordetella pertussis: variables in the in vivo priming and in vitro cytokine detection techniques affect the classification of T-cell subsets as Th1, Th2 or Th0.. Immunology.

